# Physico-Mathematical Simulation of a Homogeneous Thermal Field of Multichannel Raster Matrixes for Sensors of Oxygen

**DOI:** 10.3390/s150101404

**Published:** 2015-01-13

**Authors:** Vitalij Kotovskyi, Yurii Dzhezherya, Aleksandr Dovzhenko, Nikolaj Višniakov, Andžela Šešok

**Affiliations:** 1 Department of Applied Physics, National Technical University of Ukraine “KPI”, Peremogy av. 37, 03224 Kiev, Ukraine; E-Mails: kotovsk@kpi.ua (V.K.); dui_kpi@ukr.net (Y.D.); dovzhenkoa@ukr.net (A.D.); 2 Department of Materials Science and Welding, Vilnius Gediminas Technical University, J. Basanavičiaus str. 28, LT-03221 Vilnius, Lithuania; 3 Department of Biomechanics, Vilnius Gediminas Technical University, J. Basanavičiaus str. 28, LT-03221 Vilnius, Lithuania; E-Mail: andzela.sesok@vgtu.lt

**Keywords:** oxygenometry, electrochemical cell sensor

## Abstract

In the paper, an opportunity for the development of multichannel transcutaneous raster matrixes for sensors of oxygen on the basis of an electrochemical cell sensor is described. An analysis of the influence of heat sources on the distribution of the temperature pattern of a raster matrix for sensors of oxygen had been carried out, and their optimum configuration had been found. The application of such matrixes will enable one to obtain information about the distribution of the partial pressure of oxygen from the skin cover of the object of research in dynamics, to assess its functional health pattern in a more comprehensive way and to control the effect of possible remedial actions.

## 1. Introduction

An important indicator for the non-invasive gaining of key diagnostic data is the oxygen status of the body [1–3], which may be found from transcutaneous monitoring of the partial pressure of oxygen pO_2_ in subcutaneous tissues of biological objects.

In biomedical research, electrochemical methods that distinguish themselves for the high sensitivity and accuracy of measurements, selectivity, the linear dependence of the output signal on the analyzed substance, resistance to external mechanical impact and vibrations, small dimensions and low power consumption are the most widely used.

The key advantage of transcutaneous monitoring is that it requires considerably fewer blood samples and detects sudden changes of the above-mentioned parameter in the period between blood samplings. It is particularly important in neonatal and pediatric intensive therapy practice. In addition, monitoring ensures the representativeness of blood samples with respect to the real health status of a patient, not only his/her health status at the moment of blood sampling.

The key physical processes that take place in transcutaneous monitoring of the partial pressure of oxygen in subcutaneous tissues on the basis of an electrochemical cell of the Clark type (Clark cell) have been sufficiently explored and described in the relevant references. They include dissociation of oxyhemoglobin, oxygen transport, oxygen consumption by the cells of tissues and the sensor.

The cathodes of the cell sensors of the proposed matrix are made of platinum; the principal requirement of the anode unit is increasing the contact area and reducing the current density. For the anode unit, Ag/AgCl structures are used.

The reaction on the anode and the cathode may be described by the following equations:

$$\text{Cathode}\;\left(\text{reduction}\right)\;{\text{O}}_{\text{2}}+{2\text{H}}_{\text{2}}\text{O}+{4\text{e}}^{-}↔{\text{4OH}}^{-}$$

$$\text{Anode}\;\left(\text{oxidation}\right)\;\text{4Ag}+{\text{Cl}}^{-}↔4\text{AgCl}+{\text{4e}}^{-}$$

$$\text{General equation}\;{\text{O}}_{\text{2}}+{\text{H}}_{\text{2}}\text{O}+4\text{Ag}+{\text{Cl}}^{-}↔4\text{AgCl}+{4\text{OH}}^{-}$$

In the tests, the gas diffusion membrane that is usable in transcutaneous sensors is replaced by single-use 3–5 μm-thick polypropylene or PTFE film after its previous soaking in electrolyte solution. The film directly contacts the skin covering, which is heated up to 42 °C by the heat source integrated in the matrix. Thus, a “diffusion corridor” is formed and O_2_ from the capillaries penetrates into the cell sensors of the matrix.

When oxygen transfers from capillaries to the sensor cells of the matrix, a “diffusion corridor” is formed because of the local heating of the skin cover, and the electrochemical reaction causes an electric current of several tens of nanoamperes. The amplifier connected to the cathode measures the current and transforms it into a value proportional to the partial pressure of oxygen in the sensor/membrane interface. The said value is displayed on the monitor in mmHg.

One of the key components of equipment for non-invasive examination of the oxygen status of the body is a primary transducer of obtained diagnostic information—An electrochemical transcutaneous sensor of oxygen (TSO) [4–6]. It is a complicated multifactorial system, where the mechanism and kinetics of electrochemical processes depend considerably on the developed structures and manufacturing technologies.

In clinical practice, gas analyzers from various manufacturers, such as TCM3 Radiometer (Denmark) [7], TCO2M Respironics Inc. (USA), Hellinge (Germany), and so on, are the most widely used. However, in spite of the existence of world leaders in the production of transcutaneous gas analyzers, professionals of this sector are permanently involved in abundant research works related to various technical and metrological problems of gas analysis, such as calibration, drift prevention, analysis of the determinants of gas pressure on the surface of the skin cover, methods of production of reliable and practicable sensors, multichannel oxygenometry, and so on [4–6,8–10].

As a rule, a majority of pathologies appear in biological objects because of the disturbance of the oxygen (O_2_) supply to certain zones of tissues; this gas is transported exclusively by the erythrocytes of blood, and they are also involved in gas exchange O_2_ ↔ CO_2_ [11–13]. An analysis of scientific papers [13,14] related to problems of gas exchange in a human body shows that if pO_2_ is reduced, the overall rate of diffusion of said gas may be kept the same or increased by increasing the diffusion surface only.

From the physiological viewpoint, the most important phases of oxygen transportation are predetermined by the microvascular network and the intercapillary space. An experimental analysis of pO_2_ distribution in the specific zones requires using special, high-sensitivity microelectrodes with the minimum response time with the diameter of the working part equal to 1–5 μm, so that the standards to be set for the research equipment are raised.

The probability of the opportunity of transcutaneous pO_2_ monitoring in subcutaneous tissues is higher when the local blood flow is more intensive, as compared to the blood supply at rest.

The prerequisite for transcutaneous tests *in vivo* is ensuring of the opportunity to heat the zone of the skin cover under the test in the TSO. If the temperature of the sensor is increased from 41 °C to 45 °C, the transcutaneously-measured pO_2_ increases at each temperature step. When the temperature of the sensor equals 44 °C, the plateau is reached, and then, an increase of temperature does not cause increasing of the transcutaneous value of pO_2_, because the maximum hyperemia with vasodilatation in the zone under measurement appears.

Transcutaneous oxygenometry is classified as a method for assessing microhemodynamics, which enables obtaining information about the functional status of the vascular system, required for understanding the adequacy of the collateral blood flow, the degree of ischemia of tissues, in order to make a wise choice between conservative therapy and surgical treatment.

In addition to local hyperemia of the intact skin tissue, a number of other factors, such as the thickness of the epidermis, O_2_ consumption by the electrode, dermal respiration, and so on, are of a great importance for pO_2_ control.

For transcutaneous measurements, a very important factor is the skin covering, which plays the role of a supplemental membrane. The value of the current caused by the reduction of oxygen molecules on the sensor's cathode depends on the thickness of the membrane of the transcutaneous sensor and on the epidermis that is free of blood-vessels.

Molecules of O_2_ diffuse through the upper layers of the skin covering. However, O_2_ supply to the skin covering takes place in a way that ensures the reduction of the gradient of the said gas to zero between the dermal capillaries and the upper layer. Only when the local blood supply increases, pO_2_ measured transcutaneously on the surface of the skin increases, as well.

The development of devices on the basis of TSO with high metrological performance is an important task that may be implemented upon applying fundamentally new approaches.

## 2. Methods

The problem of diagnostics of biological objects bound with gas analysis is being settled by the development of multichannel means, namely raster matrixes for sensors of oxygen [15–17]. The application of such matrixes will enable one to obtain pO_2_, information from the skin cover of the object under research in dynamics with its subsequent processing and to extend the opportunities for exploring the functional status of biological objects.

In the course of developing such a diagnostic system, a number of technical and conceptual problems arise, and they should be settled. One of them is ensuring the same temperature conditions for the functioning of all sensors of oxygen in the raster matrix.

The optimum temperature of the skin cover that ensures the effective operation of a cell sensor of a gas analyzer is known to be *t* ≈ 43 °C; it is transferred to capillaries and ensures the formation of a “diffuse corridor”, so pO_2_ is artificially increased in the subcutaneous layer. On average, a temperature rise of 1 °C causes a rise of pO_2_ by 5% and results in the diffusion of molecular O_2_ through the epidermis and gas diffuser polypropylene membrane to the cathode of the transcutaneous sensor or oxygen.

For the purpose of safety, it is usually pointed out in medical records and operation manuals for using transcutaneous sensors that their location on a patient's body should be changed on a regular basis to reduce the risk of thermal damage to the skin. The risk of such damage depends on the temperature of the sensor, the duration of the application of the sensor and the physiological parameters, including the local perfusion, the temperature of the body and the thickness of the skin cover. As a rule, the above-described concerns newly born or small babies, because their skin cover is considerably thinner and very tender. The above-described issue is fully applicable to a multichannel raster matrix for sensors of oxygen, as well.

The task is to equip a matrix for sensors of oxygen with a plane heat source that, upon contact with the surface of a biological object, would form a zone with a homogenous distribution of temperature in the place of the disposition of the sensors. This task may be implemented in two ways. For example, each sensor may be equipped with an individual heat source and temperature measuring equipment, and then, the heating mode should be chosen in an empirical way. However, this approach is inefficient and too complicated. Therefore, we offer herein forming a plane heater that shall be a conductive surface with additional conductive accessories on it for the formation of a zone with a homogenous distribution of temperature.

The advantage of the offered multichannel raster matrix for sensors of oxygen over the existing pO_2_ control devices is the extension of the research applicability of the transcutaneous method. As distinct from discrete sensors that provide pO_2_-information for a certain point, the application of such a matrix in dynamics will enable monitoring the oxygen distribution from the surface of the skin covering (to “see” the oxygen portrait of a human in analogy to the thermal portrait obtained by an IR camera), thus causing considerable extension of the capabilities for exploring the functional status of the body.

In the future, in the course of improving the technologies for the production of such matrixes, special “smart” work clothes may be created for persons involved in extreme activities, such as biomedical research, including round-the-clock monitoring of the functional status of patients.

The establishment of blood oxygenation at various points of a limb or around a chronic ulcer is a clinical method that is often applied for detecting the danger of the critical ischemia of the limb. Transcutaneous pO_2_ monitoring is widely used in departments of vascular surgery and for equipment for diabetic foot subdivisions; it enables forecasting the outcomes of treatment very accurately and establishing, if required, the abscission location.

As a rule, a majority of transcutaneous monitors ensure measurement at a single point only (one sensor). Therefore, if measurement at several points is required, the clinicians are forced to acquire several devices or to make measurements at various points by the same device. The research opportunities of the method may be extended considerably by using multichannel equipment that, in its turn, is helpful for making more precise diagnoses, thus resulting in the selection of the best means of treatment.

For example, Radiometer Company offers a monitor that enables clinicians to make simultaneous transcutaneous measurements at six points (Monitor TCM400).

Therefore, a larger number of pO_2_ measurement points provides more information to the doctor. In this sense, the offered multichannel raster matrix for sensors of oxygen is advanced and relevant. In real-world practice, the task of the creation of similar matrixes is not fully implemented anywhere. Multichannel systems for transcutaneous gas exchange monitoring will be a breakthrough in non-invasive diagnostics.

The offered multichannel raster matrix may be used for pO_2_ control in real-time mode with the accuracy of registering the said parameter being no worse than those of existing sensors of the discrete type.

### 2.1. Technical Implementation of the Means of Multichannel Oxygenometry

**Figure 1.**
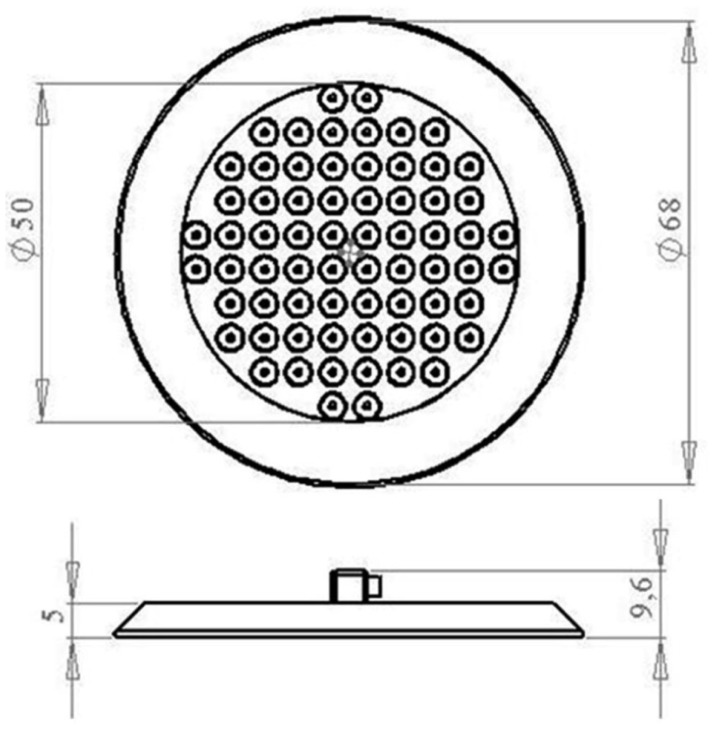
The drawing of a multichannel matrix.

**Figure 2.**
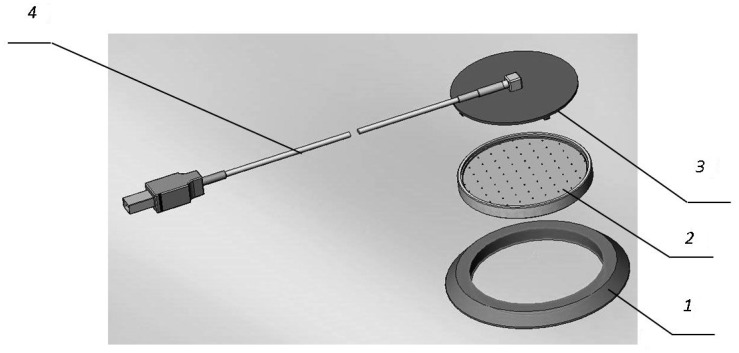
The components of the matrix: 1, a flange; 2, an anode unit; 3, a cover; 4, a connecting cable.

On the basis of an electrochemical cell sensor, the authors offer a multichannel matrix for sensors of O_2_ (Figure 1) [15]. The matrix includes 64 cell sensors and is a multichannel device with a mutual anode unit (Figure 2). The dimensions of an element are 1.5 × 1.5 mm.

**Figure 3.**
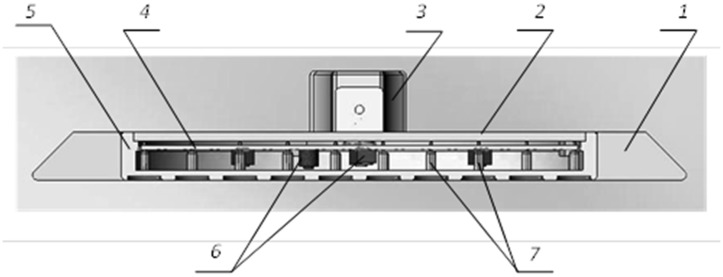
The axial section of the multichannel matrix: 1, a flange; 2, a cover; 3, a disconnecting device; 4, a control board; 5, an anode unit; 6, electronic components, thermostats; 7, the cathodes of the cell sensors.

The flange (1) is needed for protection of the anode unit and for comfortably fixing the matrix onto the skin cover. The anode unit (2) includes a board for the connection of the cathodes of the cell sensors with electronic components of the control scheme and thermostats (Figure 3). The cover (3) protects the matrix against external effects.

The control scheme of the multichannel raster matrix is involved in the polling of cell sensors, as well as multiplexing, amplification, processing and transformation of the output signal for its further processing.

### 2.2. A Heat Source

An optimum disposition of heat sources is of a great importance. The authors had discussed several versions of it.

**Figure 4.**
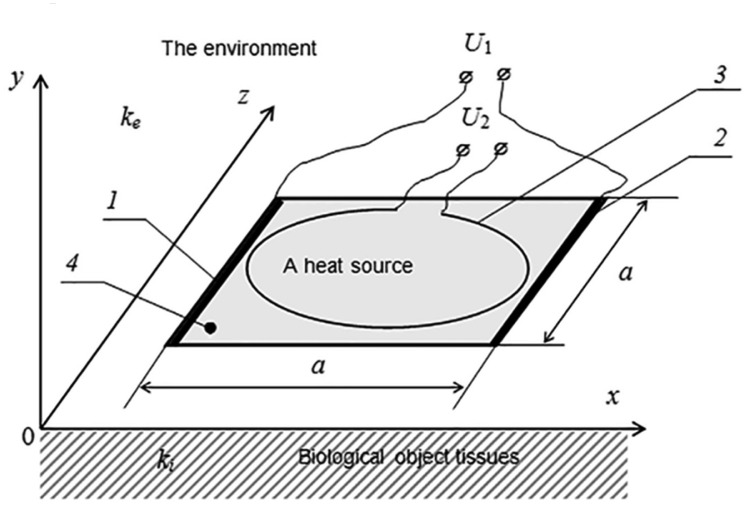
The schematic image of a combined heat source for a sensor of oxygen: 1, 2, electrodes of a high conductivity material; 3, a supplemental heat source shaped as a wire O-ring; 4, cell sensors (see Figure 3).

It is proposed that the plane heat source be made as a square-shaped structure of a conductive material. For its control, two electrodes of a high conductivity metal are fixed to the opposite sides of the square (Figure 4). The potential drop connected to the electrodes produces an electric current that is homogeneous in the plane of the conductive layer. As further calculations show, the evolved Joulean heat causes in this configuration inhomogeneous heating of the contact surface. The maximum temperature is observed in the central part. For the formation of the quasi-homogeneous distribution of temperature, it was proposed to use an additional loop-shaped electric heater. As further calculations show, the thermal field of such a heat source is the minimum in the center of the circle. Therefore, by choosing the relevant parameters of the conductive materials and the voltage of the electrodes, as well as the superposition of the heat sources, it is possible to form a large area with a quasi-homogeneous distribution of the temperature.

For establishing the optimum configuration of the heat source, we will solve the task of the definition of the stationary thermal field in the vicinity of the plane heat source situated on the border between two mediums (Figure 4). The upper medium simulates air, and the lower medium simulates the tissues of the biological object. We consider both mediums to be physically homogenous. Their heat transfer coefficients are *k_e_* and *k_i_*, correspondingly.

The plane heat source is square-shaped with sides *a*. It is made of a conductive material and may be heated by an electric current. Such a structure ensures the homogenous distribution of currents and, consequently, the homogenous density of heat sources, if two electrodes of a high conductivity material are fixed to opposite sides of the square.

In this case, the density of the homogenous current in the plane heat source is found as follows [18]:

(1) $$j=E/\rho =e_{x}U/a\rho$$

where **j** is the volumetric density of the current, **E** the voltage of the electric field in the plane electrode, *U* the voltage between the lateral electrodes, ρ the specific resistance of the material of the plane electrode and **e***_i_* the unit vectors of the coordinate system.

If a homogenous electric current Equation (1) flows in a plane conductor, the volumetric density of the heat source caused by it may be presented as follows:

(2) $$\begin{array}{l} q_{1}\left(r\right)=Q_{1}d\cdot \delta \left(z\right)\cdot \Theta \left(a/2-x\right)\cdot \Theta \left(a/2+x\right)\cdot \Theta \left(a/2-y\right)\cdot \Theta \left(a/2+y\right)\\ Q_{1}=jE=U^{2}/a^{2}\rho \end{array}$$

where *Q_1_* is the amount of heat generated by unit volume of the conductive layer, δ(*z*) the Dirac delta function, showing that the heat source is located in a plane with coordinates *z* = 0, *d* the thickness of the conductive layer and Θ(*x*) the Heaviside theta function that differs from zero and equals one, if the argument is positive.

The product of Θ functions points out that the heat sources are located in the limits of the conductive layer.

### 2.3. The Task on Definition of the Stationary Temperature Pattern

The equation for the distribution of temperatures in inhomogeneous medium with the heat source may be written as follows:

(3) $$\nabla \left(\kappa \left(r\right)\nabla T\right)=-q_{1}\left(r\right)$$

where *κ* (**r**) is the heat transfer coefficient of the medium.

Because the medium is considered piecewise continuous, the heat transfer coefficient may be expressed as follows:

(4) $$\kappa \left(r\right)=\kappa_{i}\cdot \Theta \left(z\right)+\kappa_{e}\cdot \Theta \left(-z\right)$$

After substitution of Expressions (2) and (4) into Equation (3), we obtain Laplace's equation for the thermal field and the boundary conditions for the normal components of heat flows:

(5) $$\begin{array}{l} \Delta T=0\\ \kappa_{e}{\frac{\partial T}{\partial z}|}_{z=+0}-\kappa_{i}{\frac{\partial T}{\partial z}|}_{z=-0}=-Q_{1}d\cdot \Theta \left(a/2-x\right)\Theta \left(a/2+x\right)\Theta \left(a/2-y\right)\Theta \left(a/2+y\right) \end{array}$$

where $\Delta =\frac{\partial^{2}}{\partial x^{2}}+\frac{\partial^{2}}{\partial y^{2}}+\frac{\partial^{2}}{\partial z^{2}}-$ is the Laplace operator.

The additional boundary condition is an equality of temperatures at the boundary between two mediums: ${T|}_{z=+0}={T|}_{z=-0}$ .

It is easy to make sure that the solution of the task Equation (5) (that satisfies the boundary conditions) may be expressed as follows:

(6) $$\begin{array}{l} T\left(r\right)=T_{0}+\delta T_{1}\left(r\right)\\ \delta T_{1}\left(r\right)=t_{1}\int_{-1/2}^{1/2}d\xi \int_{-1/2}^{1/2}d\eta \frac{1}{\sqrt{{\left(x/a-\xi \right)}^{2}+{\left(y/a-\eta \right)}^{2}+{\left(z/a\right)}^{2}}}\\ t_{1}=\frac{Q_{1}d\cdot a}{2\pi \left(\kappa_{e}+\kappa_{i}\right)} \end{array}$$

where *T*_0_ is a constant that defines the general level of the temperature of the medium.

The second component δ*T*(**r**) defines the impact of the plane heat source on the distribution of temperatures. The parameter *t_0_* is considered an indicative value of the heating temperature of the biological object surface zone. Choosing the parameters of the system should ensure an opportunity to control the value of *t_0_* in the range of several degrees Celsius.

If we wish to find the temperature of the surface of a biological object only, in such a case when *z* = 0, Expression (6) is integrated into elementary functions, and we obtain the following:

(7) $$\begin{array}{l} \delta T_{1}\left(x,y\right)=t_{1}\{\left(\frac{1}{2}-\frac{y}{a}\right)\ln\left(\frac{\left(1/2-x/a\right)+\sqrt{{\left(1/2-x/a\right)}^{2}+{\left(1/2-y/a\right)}^{2}}}{-\left(1/2+x/a\right)+\sqrt{{\left(1/2+x/a\right)}^{2}+{\left(1/2-y/a\right)}^{2}}}\right)-\\ \;\;\;\;\;-\left(\frac{1}{2}+\frac{y}{a}\right)\ln\left(\frac{\left(1/2-x/a\right)+\sqrt{{\left(1/2-x/a\right)}^{2}+{\left(1/2+y/a\right)}^{2}}}{-\left(1/2+x/a\right)+\sqrt{{\left(1/2+x/a\right)}^{2}+{\left(1/2+y/a\right)}^{2}}}\right)+\\ \;\;\;\;\;+\left(\frac{1}{2}-\frac{x}{a}\right)\ln\left(\frac{\left(1/2-y/a\right)+\sqrt{{\left(1/2-x/a\right)}^{2}+{\left(1/2-y/a\right)}^{2}}}{-\left(1/2+y/a\right)+\sqrt{{\left(1/2-x/a\right)}^{2}+{\left(1/2+y/a\right)}^{2}}}\right)-\\ \;\;\;\;\;-\left(\frac{1}{2}+\frac{x}{a}\right)\ln\left(\frac{\left(1/2-y/a\right)+\sqrt{{\left(1/2+x/a\right)}^{2}+{\left(1/2-y/a\right)}^{2}}}{-\left(1/2+y/a\right)+\sqrt{{\left(1/2+x/a\right)}^{2}+{\left(1/2+y/a\right)}^{2}}}\right)\} \end{array}$$

## 3. Results and Discussion

**Figure 5.**
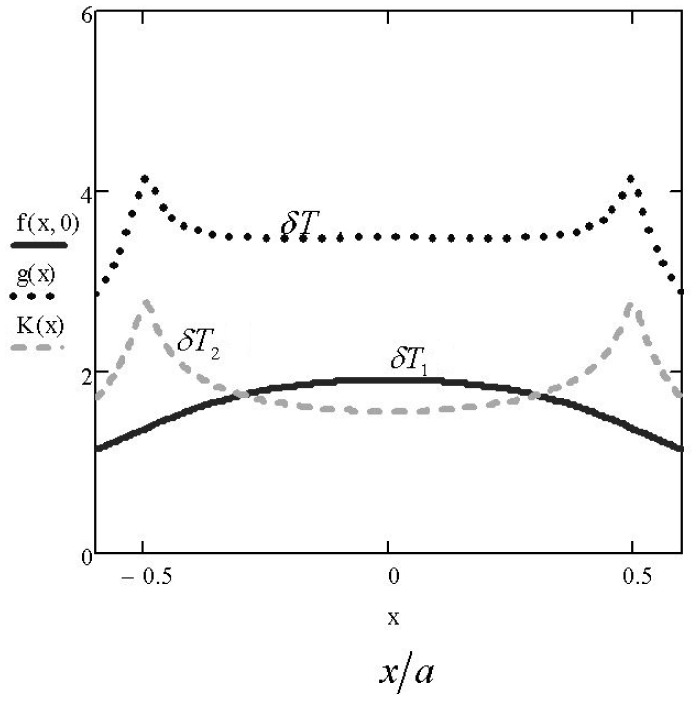
The local increase of temperature of the surface of the biological object in the location of the heaters: δ*T*_1_, the contribution of the plane heat source when *t*_1_ = 0.65 °C; δ*T*_2_, the contribution of the O-ring conductor when *t*_2_ = 0.5 °C; δ*T*, the resulting indicator of the increase in temperature; in the middle of the zone. There is a fragment with almost a homogeneous distribution of the temperature. On the ordinate axis, the graduation marks are made in degrees Celsius.

In Figure 5, the diagram of the dependence δ*T*_1_(*x, y*) on the surface of the biological object along the axis *Ox* is provided. It may be seen from the figure that the temperature of the zone planned for placing the sensors of oxygen upon using a plane heater only is highly inhomogeneous. To avoid this negative circumstance, it was possible, for example, to simulate the conductive layer according to its thickness and to make the heat source inhomogeneous. However, this method is non-technological. Therefore, we propose to place a supplemental O-ring heat source over the conductive layer (Figure 4). The impact of such a heat source on the distribution of the temperature pattern is defined by Equation (3), and the heat source is defined by the following formula:

(8) $$\begin{array}{l} q_{2}\left(r\right)=Q_{2}S\cdot \delta \left(r-a/2\right)\delta \left(z-z_{0}\right)\left(a/2\right)\cdot d\varphi \\ Q_{2}=U^{2}/\pi RaS \end{array}$$

where *U*, *R* are the voltage decrease and the total resistance of the O-ring conductor, respectively, *S* the area of the cross-section of the conductor, $r=\sqrt{x^{2}+y^{2}}$ , *φ* the angular variable of the cylindrical coordinate system and *z_0_* the distance between the plane of the O-ring conductor and the surface of the biological object.

If the distance between the plane of the O-ring conductor and the surface of the biological object is small, *i.e.*, *z*_0_ ≪ *a*, the temperature changes caused by the current flow may be approximately expressed by the following dependence:

(9) $$\begin{array}{l} \delta T_{2}\left(x,y,z\right)=t_{2}\cdot \int_{0}^{\pi }\frac{d\varphi }{\sqrt{1+{\left(2r/a\right)}^{2}-\left(4r/a\right)\cos\varphi +4{\left(z-z_{0}\right)}^{2}/a^{2}}}\\ t_{2}=\frac{Q_{2}S}{\pi \left(\kappa_{e}+\kappa_{i}\right)},\;r=\sqrt{x^{2}+y^{2}} \end{array}$$

On the surface of the biological object, if the value 4*z*_0_^2^/*a*^2^ ≪ 1 is neglected, Expression (9) shall be transformed as follows:

(10) $$\delta T_{2}\left(x,y\right)=4t_{2}K\left(2r/a\right)$$

where *K* (*r*) is the full elliptic integral.

Expression (10) is applicable when *a* − 2*r* ≫ *z*_0_. At the point *r* = *a*/2, the logarithmic singularity expressed as $\sim \ln\left(\frac{S+z_{0}^{2}}{a^{2}/4}\right)$ takes place.

**Figure 6.**
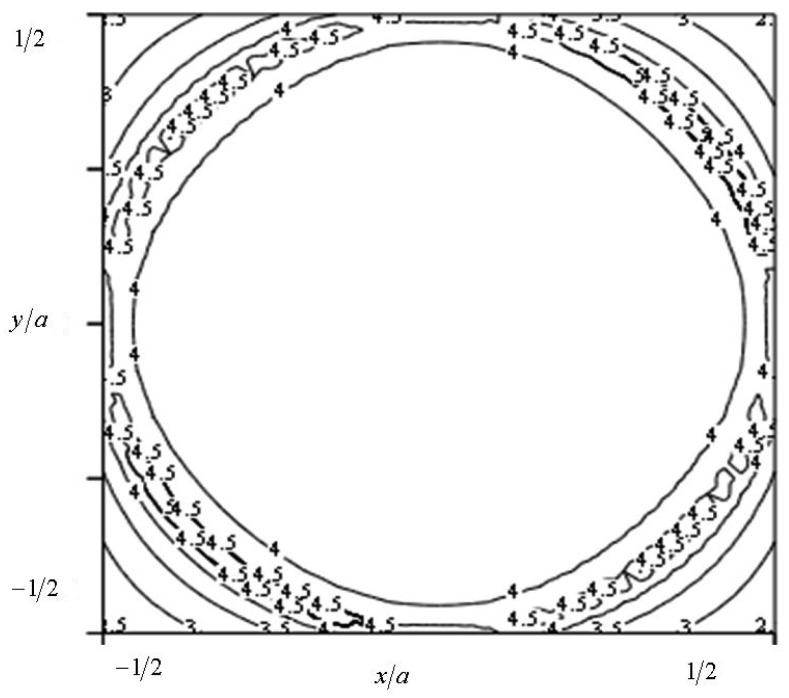
Isotherms on the surface of the biological object, in the zone of the combined heat source.

Figure 5 illustrates the contribution of the linear O-ring heat source and the results of the combined operation of the two above-mentioned heat sources. To clarify the distribution of the surface temperature pattern, the isotherms are provided in Figure 6.

It is clear from Figure 6 that the central part distinguishes itself by an almost homogeneous distribution of the temperature. Therefore, it is shown that a sufficiently homogeneous distribution of the temperature in the zone of the sensors of oxygen may be achieved by choosing the relevant geometry of the system, the conductivity of the materials and the voltage of the heat elements.

Of course, the supplemental equipment of the touchpad over the plane heat source will cause changes in the distribution of the temperature calculated in this paper. However, the specified principal approach for ensuring the homogeneity of the temperature on the surface of the skin cover will remain the same.

## 4. Conclusions

Upon striving to extend the opportunities for assessing the functional health pattern of biological objects by applying methods of transcutaneous oxygenometry, it was proposed to use electrochemical cell sensors as a primary transducer for multichannel transcutaneous raster matrixes of sensors of oxygen, which will enable one to obtain information about the distribution of the partial pressure of oxygen from the skin cover of the object of research in dynamics with its subsequent processing.

The proposed principal scheme of the spatial disposition of heat sources will ensure the homogeneous distribution of the temperature of the skin cover in the zone the oxygenometry matrix.

In addition, analytical expressions of the dependence of the temperature pattern on the geometrical and energy characteristics of the heat sources are provided.
